# Inhibition of Distinct Proline- or *N*-Acetylglucosamine-Induced Hyphal Formation Pathways by Proline Analogs in *Candida albicans*

**DOI:** 10.1155/2020/7245782

**Published:** 2020-11-17

**Authors:** Tatsuki Sato, Hisashi Hoshida, Rinji Akada

**Affiliations:** Department of Applied Chemistry, Graduate School of Sciences and Technology for Innovation, Yamaguchi University, Ube 755-8611, Japan

## Abstract

*Candida albicans* undergoes a yeast-to-hyphal transition that has been recognized as a virulence property as well as a turning point leading to biofilm formation associated with candidiasis. It is known that yeast-to-hyphal transition is induced under complex environmental conditions including temperature (above 35°C), pH (greater than 6.5), CO_2_, *N*-acetylglucosamine (GlcNAc), amino acids, RPMI-1640 synthetic culture medium, and blood serum. To identify the hyphal induction factor in the RPMI-1640 medium, we examined each component of RPMI-1640 and established a simple hyphal induction condition, that is, incubation in L-proline solution at 37°C. Incubation in GlcNAc solution alone, which is not contained in RPMI-1640, without any other materials was also identified as another simple hyphal induction condition. To inhibit hyphal formation, proline and GlcNAc analogs were examined. Among the proline analogs used, L-azetidine-2-carboxylic acid (AZC) inhibited hyphal induction under both induction conditions, but L-4-thiazolidinecarboxylic acid (T4C) specifically inhibited proline-induced hyphal formation only, while *α*-*N*-methyl-L-proline (mPro) selectively inhibited GlcNAc-induced hyphal formation. Hyphal formation in fetal bovine serum was also inhibited by AZC or T4C together with mPro without affecting the proliferation of yeast form. These results indicate that these proline analogs are ideal inhibitors of yeast-to-hyphal transition in *C*. *albicans*.

## 1. Introduction

*Candida albicans* is an opportunistic fungal pathogen that commonly exists as a benign member of the human microbiome. Immunosuppression can predispose an individual to infection, enabling this fungus to initiate and develop a spectrum of pathologies, including superficial mucocutaneous or even life-threatening invasive infections [1, 2]. Over the past decade, Candida infections have increased dramatically with the rise in AIDS, cancer, and bone marrow transplant patients.

As a pleomorphic organism, *C. albicans* can assume at least three distinct morphologies: yeast like, pseudohyphae, and true hyphae, where the latter two are commonly referred to as filamentous morphologies [3–6]. Morphogenesis has long been a central focus of research in *C*. *albicans* because of links between each of these morphologies and important host fungal interactions. Traditionally, filamentous forms of *C*. *albicans* were considered pathogenic, whereas the yeast form was primarily viewed as commensal. Therefore, inhibition of yeast-to-hyphal transition is a promising target to prevent candidiasis. In this direction, nontoxic small molecules that inhibit *C. albicans* yeast-to-hyphal transition and hyphal growth were screened for the development of antifungal agents [7–10].

To elucidate the yeast-to-hyphal transition mechanism in *C. albicans*, various hyphal induction conditions were investigated. Numerous host-related environmental factors, including mammalian physiological temperature (37°C), CO_2_, neutral-alkaline pH, serum, *N*-acetylglucosamine (GlcNAc), nitrogen starvation, and a discrete set of amino acids have been shown to be involved in the transition mechanism [6, 11–21]. In addition, incubation at 37°C in yeast, peptone, and dextrose (YPD) medium plus serum, YPD medium plus GlcNAc, Spider medium, Lee's medium, and RPMI-1640 medium have been used as common experimental conditions for inducing hyphal formation in *C. albicans* [6, 11–21]. However, these conditions include excessive complexity, such as carbohydrates, peptides, amino acids, vitamins, minerals, and/or buffer components as well as hyphal inducers, such as amino acids, GlcNAc, and serum. Therefore, in addition to hyphal inducers, these supplemental components may complement or play an important role in hyphal induction and elongation.

In this study, we hypothesized that simplification of such a complex hyphal-inducing condition is important to understand the yeast-to-hyphal transition mechanism. To address this hypothesis, we first took the approach of decomposing the RPMI-1640 medium recipe, and the hyphal-inducing condition was simplified to the utmost limit. The results showed that a small amount of L-proline is an effective component of hyphal formation. This result allowed us to examine proline analogs as inhibitors of hyphal induction. Proline analogs inhibited proline- or GlcNAc-induced hyphal formation. Therefore, multiple uses of these analogs, acting on different hyphal induction pathways, will potentially become effective antihyphal drugs.

## 2. Materials and Methods

### 2.1. Yeast Strain and Hyphal Induction Conditions

*C*. *albicans* JCM1542 strain was provided by the RIKEN BioResource Research Center through the National BioResource Project of the MEXT/AMED, Japan. *C*. *albicans* cells were cultured in Sabouraud medium (peptone 10 g/L, glucose 20 g/L, and yeast extract 5 g/L) in a rotary shaker (150 rpm) at 30°C overnight. For hyphal induction, the overnight culture was washed with sterile MilliQ water (water), and the cells were suspended in RPMI-1640 medium (Nakalai Tesque: 30264, Kyoto, Japan). A volume of 0.1 mL of adjusted cells (final 1 × 10^5^ cells/mL) was inoculated into 24-well plates containing RPMI-1640 (final volume 0.5 mL/well) at 37°C or 30°C for 24 h without shaking. Then, hyphal length was measured manually from the photo of microscopic images.

For the screening experiments of hyphal induction components, glucose solution (final concentration 2 mg/mL), 50x RPMI-1640 amino acid solution (final concentration 1x, Sigma-Aldrich: R7131, St. Louis, MO), 100x RPMI-1640 vitamin solution (final concentration 1x, Sigma-Aldrich: R7256), or inorganic salts (final concentrations: 100 mg/L Ca(NO_3_)_2_, 50 mg/L MgSO_4_, 400 mg/L KCl, and 6000 mg/L NaCl) whose pH were adjusted to 6.5–7.0 with 5 M HCl or 10 M NaOH and filtered for sterilization were mixed with the diluted yeast cells (final 1 × 10^5^ cells/mL) such that the total volume is 0.5 mL in a well of 24-well plate. The plate was incubated at 37°C for 24 h.

For the amino acid screening assay, the experiment was conducted in the same way as described above except for the addition of each amino acid. The following amino acids were dissolved in water: L-alanine, L-cysteine, L-aspartic acid, L-glutamic acid, L-phenylalanine, glycine, L-histidine, L-methionine, L-asparagine, L-glutamine, L-arginine, L-serine, L-valine, L-isoleucine, L-threonine, L-hydroxyproline, and L-proline. The pH was adjusted to 6.5–7.5 with 5 M HCl or 10 M NaOH, and filtered for sterilization. The final concentrations of each amino acid are shown in Table 1. All materials were purchased from Wako Pure Chemical Corporation (Osaka, Japan).

### 2.2. Hyphal Induction with L-Proline, GlcNAc, and Fetal Bovine Serum (FBS)

For hyphal induction in L-proline, *N*-acetylglucosamine (GlcNAc; Wako Pure Chemical Corporation: 011-12182, Osaka, Japan), and fetal bovine serum (FBS; Thermo Fisher Scientific, Waltham, MA), yeast cells (final 1 × 10^5^ cells/mL) were transferred into each well of 96-well plates (final volume 0.2 mL/well) and incubated with different concentrations of L-proline and GlcNAc at 37°C for 24 h. Plates with FBS were incubated at 37°C for 4 h.

### 2.3. Search for Hyphal Formation Inhibitors

To evaluate the inhibitory effect of the selected chemicals, 0.1 mL of the adjusted cells (final 1 × 10^5^ cells/mL) were inoculated into wells containing L-proline or GlcNAc in 24-well plates. Then, 0.1 mL of each chemical was added. The final concentrations of L-proline, GlcNAc, and chemicals were 10 mM, 5 mM, and 20 mM, respectively. The chemicals (100 mM solutions) used were L-4-thiazolidinecarboxylic acid (T4C; Sigma-Aldrich: T27502), tetrahydro-2-furoic acid (THFA; Sigma-Aldrich: 341517), L-pipecolic acid (Tokyo Chemical Industry Co., Ltd., Tokyo, Japan (TCI): P1404), L-azetidine-2-carboxylic acid (AZC; TCI: A1043), L-pyroglutamic acid (TCI: P0573), *α*-*N*-methyl-L-proline (mPro; TCI: M2077), L-lactic acid (TCI: L0165), and *N*-trifluoroacetyl-D-glucosamine (TCI: T0973). The pH of each chemical solution was adjusted to 6.5–7.5 using 10 M NaOH and filtered for sterilization prior to addition, and the mixture was incubated at 37°C for 24 h. As a control, 10 mM L-proline or 5 mM GlcNAc without chemicals was also incubated.

Hyphal inhibitory effects of AZC, T4C, and mPro were determined in a 96-well microplate. *C*. *albicans* cells were diluted to a final concentration of 1 × 10^5^ cells/mL in a solution with 10 mM L-proline or 5 mM GlcNAc with different concentrations of AZC, T4C, or mPro. The final volume was kept at 200 *μ*L in each well. The microplate was incubated at 37°C for 24 h. The numbers of both yeast and hyphal cells in photomicrographs were counted, and the percentage of hyphal cells were calculated as the number of hyphal cells divided by the number of total cells. At least 30 cells were counted. All the experiments were done in triplicates.

Hyphal inhibitory effects of 20 mM AZC, 20 mM T4C, and 20 mM mPro in the presence of FBS were examined in the same way as described above except for the addition of different concentrations of FBS.

Hyphal inhibitory effects of AZC and simultaneous addition of T4C and mPro were examined in FBS. In brief, yeast cells (final 10^5^ cells/mL in water) were transferred into each well of the 96-well plates and incubated with AZC, T4C, or mPro (final concentrations of AZC, T4C, and mPro: 20, 10, and 5 mM) and different concentrations of FBS (final concentrations of 50, 25, 12.5, or 6.3%) at 37°C for 4 h. The numbers of both yeast and hyphal cells in photomicrographs were counted, and the percentage of hyphal cells was calculated as the number of hyphal cells divided by the number of total cells. At least 30 cells were counted. All the experiments were done in triplicates.

### 2.4. Evaluation of the Inhibitory Effect on Cell Proliferation

To assess the inhibitory effect of the compounds on cell proliferation, yeast growth was analyzed using a TVS062CA Biophotorecorder (Toyo Seisakusho, Ltd., Chiba, Japan). *C. albicans* cells were washed with water and 1 × 10^5^ cells/mL were inoculated in 5 mL Sabouraud medium with or without chemicals. The cell suspensions were incubated at 30°C with shaking at 70 rpm, and the optical density (OD) at 660 nm was recorded every 15 min.

## 3. Results

### 3.1. Determination of Hyphal Induction Factor in RPMI-1640 Medium

**Figure 1:**
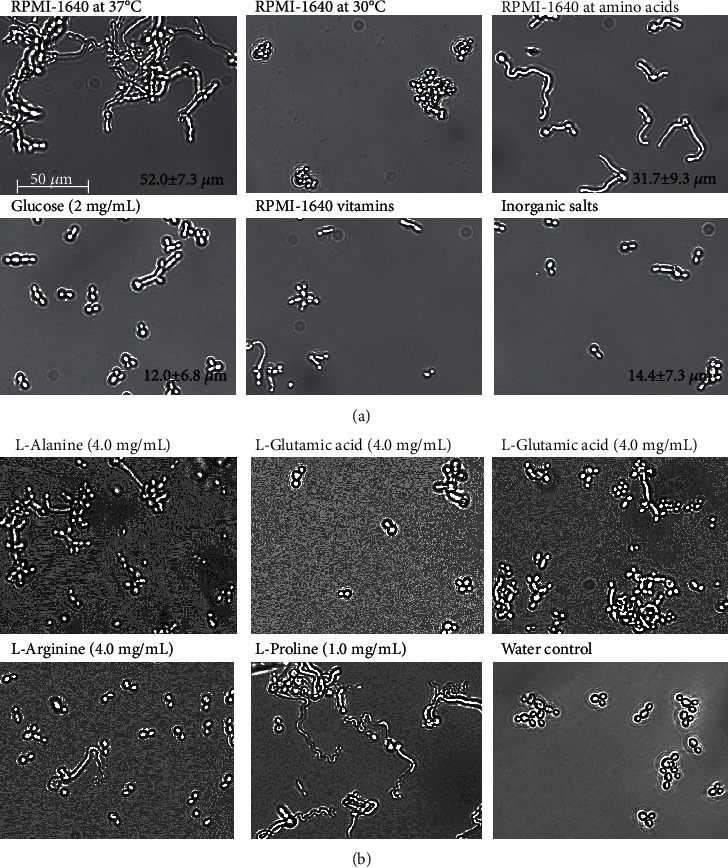
(a) Morphology of *C*. *albicans* and hyphal length under different hyphal-inducing conditions. Standard deviations are shown for each sample. (b) Effect of amino acids in the RPMI-1640 medium. Cells of *C*. *albicans* (10^5^ cells/mL) were incubated at 37°C except for RPMI-1640 at 30°C for 24 h. RPMI-1640 amino acids and vitamins were purchased from Sigma-Aldrich and used at 1x concentration. Inorganic salts were 100 mg/L Ca(NO_3_)_2_, 50 mg/L MgSO_4_, 400 mg/L KCl, and 6000 mg/L NaCl. The scale bar was applied to all images.

To identify key environmental factors and components of hyphal induction in *C*. *albicans*, we first confirmed whether the *C*. *albicans* JCM1542 strain transformed to hyphal form in the RPMI-1640 medium, a completely chemically defined medium that is known as the basic hyphal induction condition [6, 11–13]. After cells were incubated in RPMI-1640 medium at 37°C for 24 h, *C*. *albicans* showed hyphal morphology (Figure 1(a)). Hyphal formation was observed at 37°C; however, when cells were incubated at 30°C for 24 h, hyphal formation was not observed (Figure 1(a)). These results revealed that RPMI-1640 medium could induce hyphal formation in *C*. *albicans* JCM1542 strain and that the temperature of 37°C was a critical factor for hyphal induction in RPMI-1640 medium.

Since we confirmed that the RPMI-1640 medium induced hyphal formation, we searched for hyphal induction components in the RPMI-I640 medium. The RPMI-1640 medium composition was classified into four categories: carbohydrates, amino acids, vitamins, and inorganic salts. Cells were incubated with 2 mg/mL glucose, 1x RPMI-1640 amino acid solution, 1x RPMI-1640 vitamin solution, or inorganic salts at 37°C for 24 h. Results showed that the RPMI-1640 amino acid solution induced hyphal formation, although hyphal length (31.7 ± 9.3 *μ*m) was shorter than the RPMI-1640 medium 52.0 ± 7.3 *μ*m (Figure 1(a)). On the other hand, 2 mg/mL glucose and RPMI-1640 vitamin solution induced very short hyphae (12.0 ± 6.8 and 14.4 ± 7.3 *μ*m), whereas inorganic salts did not induce hyphae. These results indicated that critical components for hyphal induction in the RPMI-1640 medium are included in the RPMI-1640 amino acid solution (Figure 1(a)).

Next, to determine which amino acid induces hyphal formation, cells were incubated with individual amino acids included in the RPMI-1640 medium at 37°C for 24 h. As shown in Figure 1(b) and Table 1, L-proline showed more than 50% hyphal formation, whereas L-alanine, L-glutamic acid, L-phenylalanine, and L-arginine showed fewer hyphal formation than L-proline (less than 25% hyphal formation). Hyphae were not present in the other amino acids (Table 1). These results showed that L-proline induced the largest number of hyphal formation in the RPMI-1640 medium.

**Figure 2:**
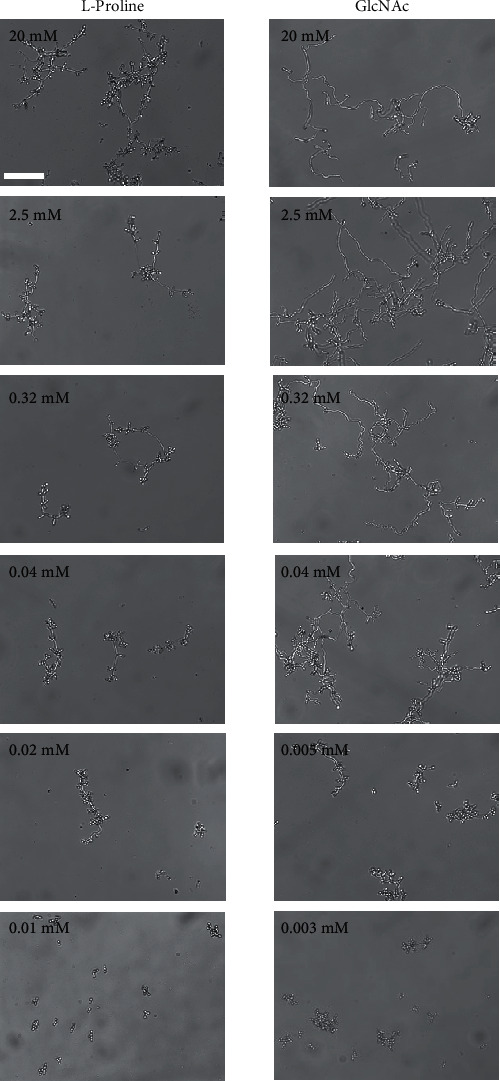
Hyphal induction in different concentrations of L-proline and GlcNAc. Cells of *C*. *albicans* (10^5^ cells/mL) were incubated with various concentrations of L-proline or GlcNAc at 37°C for 24 h. No hyphal formation was observed in 0.01 mM L-proline and 0.003 mM GlcNAc. The scale bar (100 *μ*m) was applied to all images.

Next, the minimal hyphal induction concentration of L-proline was examined. Cells were incubated with different concentrations of L-proline at 37°C for 24 h. Hyphal formation was observed in 0.02 mM (Figure 2) but not in less than 0.01 mM L-proline. As 0.17 mM L-proline is included in the RPMI-1640 medium, this result revealed that the RPMI-1640 medium contains enough L-proline to induce hyphal formation.

Subsequently, we searched for further simple hyphal-inducing conditions other than L-proline. GlcNAc is widely known as a hyphal inducer in *C*. *albicans*. It is generally used with other nutrients, such as carbohydrates, nitrogen sources (peptone, yeast extract, or amino acids), and vitamins [13, 19–21]. Therefore, we examined whether GlcNAc alone at low concentrations could also induce hyphal formation by itself. Figure 2 shows that a single addition of GlcNAc could also induce hyphal formation at a minimal concentration of 0.005 mM, which is almost 100-fold lower than the usual concentration (2.5-5 mM) [13, 19–21]. From these results, we established two different types of simplified hyphal-inducing conditions.

### 3.2. Search for Hyphal Formation Inhibitors

**Figure 3:**
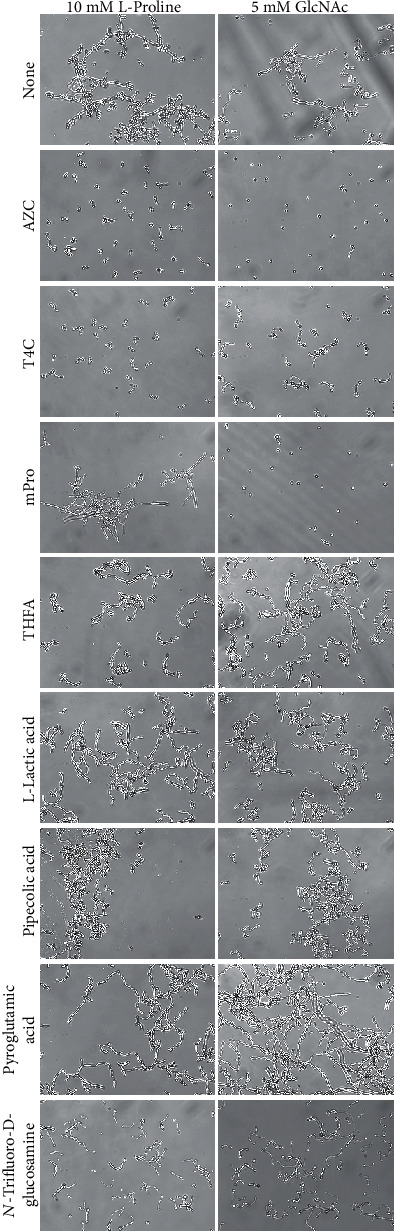
Effect of proline analogs, proline metabolism inhibitors, and GlcNAc analog on 10 mM L-proline- or 5 mM GlcNAc-induced hyphal formation. Cells of *C*. *albicans* (10^5^ cells/mL) with various compounds (final 20 mM) were incubated at 37°C for 24 h. The scale bar (100 *μ*m) was applied to all images.

As L-proline was determined as a critical hyphal inducer in the RPMI-1640 medium, we hypothesized that proline analogs and proline metabolism inhibitors could be potential antihyphal drug candidates. We selected six proline analogs, THFA [22–25], T4C [14, 15, 26, 27], L-pipecolic acid [22, 24], AZC [14, 15, 26, 28], L-pyroglutamic acid, and *α*-*N*-methyl-L-proline (mPro), and one known proline metabolism inhibitor, L-lactic acid [23, 28, 29]. In addition, *N*-trifluoroacetyl-D-glucosamine, which is a GlcNAc analog, was also examined. Cells were incubated in 10 mM L-proline or 5 mM GlcNAc with each chemical compound (final concentration 20 mM) at 37°C for 24 h, and their morphologies were assessed. As shown in Figure 3, AZC strongly inhibited hyphal formation under both conditions and T4C showed strong hyphal inhibitory effect in 10 mM L-proline but slight inhibition in 5 mM GlcNAc. Interestingly, mPro completely inhibited hyphal formation in 5 mM GlcNAc but did not inhibit it in 10 mM L-proline. Other compounds did not inhibit hyphal induction in either condition (Figure 3).

Thus, AZC, T4C, and mPro showed different hyphal inhibitory effects in the two different simple hyphal-inducing conditions, even though they are categorized as proline analogs.

Next, minimal hyphal inhibitory effects of AZC, T4C, and mPro were determined in the condition that showed hyphal inhibition. Cells were cultured in 10 mM L-proline or 5 mM GlcNAc with different concentrations of AZC, T4C, or mPro at 37°C for 24 h, and their morphologies were assessed.  Table 2 shows that the minimal concentration of AZC was determined to be 10 mM (hyphal formation 67.3 ± 3.1%) in proline and 0.6 mM (38.5 ± 6.7%) in GlcNAc. On the other hand, the minimal concentration of T4C was 20 mM in proline (40.0 ± 6.1%) and the minimal concentration of mPro was 10 mM (71.3 ± ±5.5%) in GlcNAc.

### 3.3. Hyphal Inhibitory Effect of Proline Analogs under Serum Conditions

**Figure 4:**
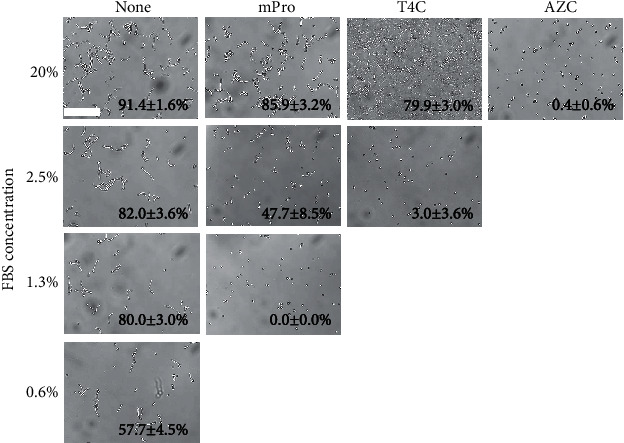
Effect of proline analogs on FBS-induced hyphal formation. Cells of *C*. *albicans* (10^5^ cells/mL) with various compounds (20 mM) were incubated at 37°C for 4 h. All assays were triplicated, and at least 30 cells were counted for hyphal formation percentage with standard deviations. The scale bar (100 *μ*m) was applied to all images.

Because proline and glucosamine are included in serum [30, 31], we hypothesized that proline and/or GlcNAc in serum may lead to hyphal formation. We examined whether AZC, T4C, and mPro could suppress yeast-to-hyphal transition in the presence of FBS. Cells were cultured in different concentrations of FBS with or without AZC, T4C, or mPro (final concentration 20 mM) at 37°C for 4 h, and their morphologies were assessed. As shown in Figure 4, the minimal hyphal induction concentration of FBS was observed at 0.6% (57.7 ± 4.5% hyphal formation). On the other hand, AZC completely inhibited hyphal formation even in 20% FBS (0.4 ± 0.6% hyphal formation). T4C inhibited hyphal induction in less than 2.5% FBS (3.0 ± 3.6% hyphal formation), and mPro inhibited in less than 1.3% FBS (0.0 ± 0.0% hyphal formation).

**Figure 5:**
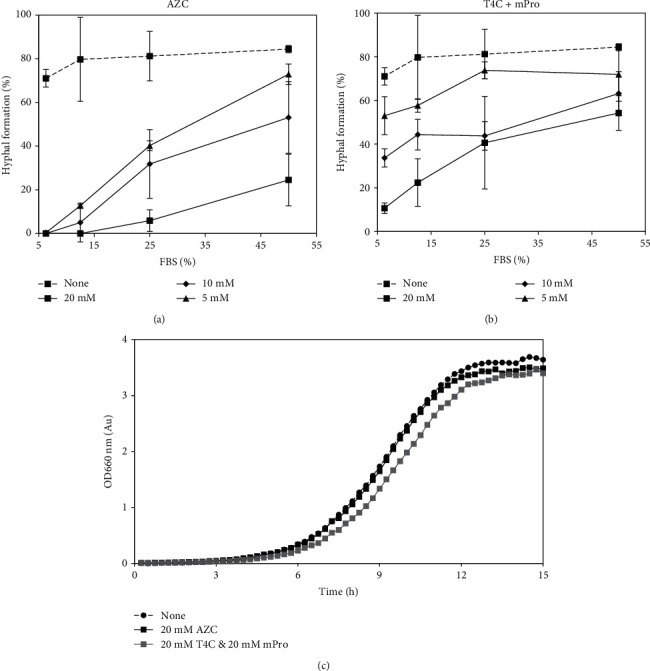
Effect of proline analogs. (a) Effect of AZC on different concentration of FBS yeast-to-hyphal transition. (b) Effect of simultaneous addition of T4C and mPro on yeast-to-hyphal transition in different concentration of FBS. *C*. *albicans* (10^5^ cells/mL) with various compounds (final concentration was 20 mM) were incubated at 37°C for 4 h. At least 30 cells were counted and all assays were triplicated. Standard deviations are shown for each sample. (c) Effect of proline analogs on yeast form growth of *C*. *albicans*. *C*. *albicans* cells (10^5^ cells/mL) in Saboraoud's medium were cultured with 20 mM AZC, 20 mM T4C, and 20 mM mPro, or without at 30°C and OD 660 nm was recorded every 15 minutes by biophotorecorder.

Next, the hyphal inhibitory effects of simultaneous addition of T4C and mPro were examined because both proline and GlcNAc pathways are blocked by these inhibitors. Cells were incubated with 20 mM AZC, 10 mM AZC, or 5 mM AZC and a combination of 20 mM T4C and 20 mM mPro, 10 mM T4C and 10 mM mPro, or 5 mM T4C and 5 mM mPro in different concentrations of FBS.  Figure 5(a) shows that 20 mM AZC strongly inhibited hyphal formation in FBS (less than 30% hyphal formation in 50% FBS and more than 50% inhibition in 50% FBS by 10 mM AZC). The combination of T4C and mPro inhibited hyphal formation even at higher concentrations of FBS (Figure 5(b)). Moreover, cell growth in the yeast form was evaluated in the presence of AZC, T4C, and mPro.  Figure 5(c) shows that all compounds did not significantly affect cell growth in the yeast form.

## 4. Discussion

### 4.1. *Candida* Hyphal Induction Factors

In previous studies, hyphal induction was usually conducted under complex conditions such as the RPMI-1640 medium, Lee's medium, Spider medium, YPD plus serum, and YPD plus GlcNAc [12–21]. All these conditions and media include multiple factors and undefined components. In this study, we first simplified hyphal-inducing conditions to the utmost limit. We think that incubation in L-proline solution at 37°C is the simplest hyphal induction condition. Our study also demonstrated that proline could induce hyphal formation at much lower concentrations (0.02 mM) than normal hyphal-inducing concentrations of 10–50 mM [14–19]. Previous studies reported that L-proline stimulates hyphal induction; however, other components, such as carbohydrates, nitrogen sources, vitamins, buffer components, and/or inorganic salts, were included in addition to 10-50 mM proline [14–19]. Moreover, it was reported that arginine induced hyphal formation along with proline [18]; other studies demonstrated that alanine, arginine, histidine, isoleucine, and lysine induced hyphal formation [16, 17]; however, these amino acids did not show hyphal formation in our simple condition. Therefore, we hypothesized that other nutrients may generate a synergistic effect on hyphal formation. Proline is the only amino acid with a secondary amine, but all others have a primary amine. This difference may be recognized by the metabolic pathways, either leading to hyphal formation or yeast form growth. L-proline induced hyphal formation, but the hyphal length was shorter than that induced by the RPMI-1640 medium (Figure 1(a)). This suggested that other nutrients contained in the RPMI-1640 medium support hyphal elongation. Therefore, L-proline may be a suitable nutrient for initiating transformation from yeast to the hyphal form although it is not an effective nutrient for cell growth.

In addition to proline, we identified that incubation in the GlcNAc solution at 37°C is another simple hyphal induction condition that could induce hyphal formation by itself at a much lower concentration (0.005 mM) than known concentrations, 2.5–5 mM [13, 19–21, 26]. This may be the first report demonstrating that only a small amount of proline (0.02 mM) alone or GlcNAc (0.005 mM) alone is sufficient to induce hyphal formation, although it may not be enough in providing nutrients for cell growth.

### 4.2. Hyphal Formation Inhibitors

The emergence of resistance to therapeutic agents is of increasing concern, which is why investigations on the antifungal potential of new molecules are highly relevant [32–36]. Compounds affecting hyphal formation have been focused on for searching the drug targets in *C*. *albicans* because the filamentous forms are considered pathogenic [7–11]. For example, virulence factors such as proteolytic and lipolytic enzymes are secreted from *C*. *albicans* when it switches from the yeast form to an invasive hyphae morphotype [3, 7–11]. Moreover, genetically defined mutant strains locked in the yeast morphology, and therefore unable to be filamentous, were avirulent in a murine model of invasive *C*. *albicans* infection [37]. Subsequent studies using regulatable strains in which morphogenetic conversions could be controlled both in vitro and in vivo provided compelling evidence for the role of filamentation in the progression to active infection [38, 39].

In this study, L-proline and GlcNAc are hyphal induction factors that could induce hyphal formation by their single addition into water. Thus, these results enabled us to focus on searching for chemical compounds possessing hyphal inhibitory effects involving L-proline and GlcNAc; we found that AZC, T4C, and mPro inhibited hyphal formation in the presence of proline and/or GlcNAc (Figure 3). AZC inhibited hyphal formation in both proline and GlcNAc (Table 2). Previous studies reported that hyphal induction by L-proline was linked to hyphal induction by GlcNAc through chitin construction, and L-proline uptake was inhibited in the presence of higher concentrations of GlcNAc without any impact on hyphal formation [40]. Thus, the correlation between L-proline and GlcNAc is known during germ tube formation. T4C inhibited hyphal formation in proline but did not effectively inhibit it in GlcNAc. In contrast, mPro inhibited hyphal formation in GlcNAc but did not inhibit it in proline (Table 2). Proline- and GlcNAc-induced hyphal induction pathways have been known to be distinct [18, 21]. However, AZC action suggested that the two pathways may be merged somewhere related to the metabolism of both proline and GlcNAc.

### 4.3. Inhibition of Hyphal Formation in the Presence of FBS

Serum is one of the most potent inducers of hyphal development in *C*. *albicans* [6, 11–13]. However, the effect of the serum is complex. We hypothesized that proline and GlcNAc are important components of serum for hyphal induction. Therefore, AZC, T4C, and mPro were examined to determine whether they could effectively suppress yeast-to-hyphal transition in the presence of FBS. AZC (20 mM) inhibited hyphal formation in 20% FBS (Figure 4). In terms of T4C, hyphal inhibition was observed at less than 2.5% FBS (Figure 4). It was considered that both proline and GlcNAc exist at a sufficient concentration to induce hyphal formation at more than 2.5% FBS; therefore, T4C could not inhibit hyphal formation at this concentration. However, when serum was diluted to less than 2.5% concentration, the hyphal induction effect in GlcNAc may be negligible and proline accounts for a large percentage of hyphal induction. With mPro, hyphal inhibition was observed at less than 1.3% FBS and had a lower inhibitory effect than T4C (Figure 4). This result also may suggest that both proline and GlcNAc contribute to hyphal induction in serum, and proline accounts for a large percentage of hyphal induction at low serum concentrations. It is also reported that concentrations of proline in serum were 0.13-0.63 mM [30]. The hyphal inhibitory effect of simultaneous addition of T4C and mPro was examined at high FBS concentrations (25 and 50%). These data clearly showed that both AZC and the combination of T4C and mPro could block hyphal formation at high concentrations of FBS (Figures 5(a) and 5(b)). It is also suggested that inhibition of two routes, proline and GlcNAc, is important to control hyphal formation in *C*. *albicans*. Thus, these results suggested that proline and GlcNAc play main roles in the hyphal induction in FBS, probably in human body fluid.

## 5. Conclusions

In this study, the complex hyphal-inducing conditions were simplified to the utmost limit; proline and GlcNAc were found to show hyphal induction by their individual addition into water at low concentration. Moreover, it was suggested that both proline and GlcNAc contribute to hyphal induction by the serum. Since AZC and the combination of T4C and mPro could inhibit hyphal formation in the presence of serum without affecting yeast form cell growth, it may confer less selective pressure for the occurrence of resistance [35, 36]. Thus, our study showed that simplification of hyphal-inducing conditions is useful for understanding yeast-to-hyphal transition mechanisms in *C*. *albicans,* and also AZC and a combination of T4C and mPro may be promising candidates for antihyphal drugs.

## Figures and Tables

**Table 1:** Hyphal formation ability with amino acid in *C*. *albicans*.

Amino acid	Concentration (mg/mL)	Hyphal formation
L-alanine	4.0	+
L-cysteine	0.4	-
L-aspartic acid	4.0	-
L-glutamic acid	4.0	+
L-phenylalanine	2.0	+
Glycine	4.0	-
L-histidine	0.4	-
L-methionine	0.4	-
L-asparagine	1.4	-
L-glutamine	2.0	-
L-arginine	4.0	+
L-serine	2.0	-
L-isoleucine	0.2	-
L-threonine	0.2	-
L-hydroxyproline	2.0	-
L-proline	1.0	+++
Water control		-

-: <5% hyphal formation; +: >5% to <25% hyphal formation;++: >25% to <50% hyphal formation; +++: >50% hyphal formation.

**Table 2:** Percentage of hyphal formation induced by proline or GlcNAc in the presence of proline analogs in *C. albicans*.

Concentration of proline analogs (mM)	10 mM L-proline			5 mM GlcNAc		
	AZC	T4C	None	AZC	mPro	None
20	19.0 ± 4.0	40.0 ± 6.1	100.0	0.0	17.0 ± 4.4	100.0
10	67.3 ± 3.1	100.0		0.0	71.3 ± 5.5	
5	100.0	100.0		0.0	100.0	
2.5	100.0	100.0		7.0 ± 3.5	100.0	
1.3	100.0	100.0		11.7 ± 2.3	100.0	
0.6	100.0	100.0		38.5 ± 6.7	100.0	

At least 30 cells were counted, and all assays were performed in triplicate. Standard deviations are shown for each sample.

## Data Availability

The data used to support the findings of this study are available from the corresponding author upon request.
